# Prognostic and predictive values of long non-coding RNA *LINC00472* in breast cancer

**DOI:** 10.18632/oncotarget.3287

**Published:** 2015-03-25

**Authors:** Yi Shen, Dionyssios Katsaros, Lenora W. M. Loo, Brenda Y. Hernandez, Clayton Chong, Emilie Marion Canuto, Nicoletta Biglia, Lingeng Lu, Harvey Risch, Wen-Ming Chu, Herbert Yu

**Affiliations:** ^1^ Cancer Epidemiology Program, University of Hawaii Cancer Center, Honolulu, Hawaii, USA; ^2^ Department of Surgical Sciences, Azienda Ospedaliero-Universitaria, Città della Salute, S. Anna Hospital, Turin, Italy; ^3^ Department of Surgical Sciences, University of Turin, Torino, Italy; ^4^ Department of Chronic Disease Epidemiology, Yale School of Public Health, New Haven, Connecticut, USA; ^5^ Cancer Biology Program, University of Hawaii Cancer Center, Honolulu, Hawaii, USA

**Keywords:** lincRNA, breast, LINC00472

## Abstract

*LINC00472* is a novel long intergenic non-coding RNA. We evaluated *LINC00472* expression in breast tumor samples using RT-qPCR, performed a meta-analysis of over 20 microarray datasets from the Gene Expression Omnibus (GEO) database, and investigated the effect of *LINC00472* expression on cell proliferation and migration in breast cancer cells transfected with a *LINC00472*-expressing vector. Our qPCR results showed that high *LINC00472* expression was associated with less aggressive breast tumors and more favorable disease outcomes. Patients with high expression of *LINC00472* had significantly reduced risk of relapse and death compared to those with low expression. Patients with high *LINC00472* expression also had better responses to adjuvant chemo- or hormonal therapy than did patients with low expression. Results of meta-analysis on multiple studies from the GEO database were in agreement with the findings of our study. High *LINC00472* was also associated with favorable molecular subtypes, Luminal A or normal-like tumors. Cell culture experiments showed that up-regulation of *LINC00472* expression could suppress breast cancer cell proliferation and migration. Collectively, our clinical and *in vitro* studies suggest that *LINC00472* is a tumor suppressor in breast cancer. Evaluating this long non-coding RNA in breast tumors may have prognostic and predictive value in the clinical management of breast cancer.

## INTRODUCTION

Breast cancer is the most common female malignancy, annually accounting for more than a million new diagnoses worldwide [1]. Although breast cancer has been studied extensively for decades, mechanisms of tumor progression have remained largely elusive. Tumor heterogeneity is a major challenge to understanding the disease. Breast cancer varies not only from patient to patient, but also within the same tumor. The disease evolves over time and changes in response to chemo- and hormonal therapies [2–5]. The unstable genome of tumor cells is partially responsible for these changes [3, 6, 7]. Many studies have been conducted to search for disease features that can help to predict disease outcome and treatment response, but no robust tumor markers have yet been identified.

Proteins are considered to be the major molecules carrying out essential biologic actions [8], yet only 2% of the human genome contains the codes for proteins. Most genomic sequences are now understood to be transcribed, though without translation capability [9]. These non-protein coding transcripts are involved in many biologic processes and cellular activities. Recently, long non-coding RNAs (lncRNAs) were recognized as a new class of non-coding RNAs with important biologic functions [10–16]. LncRNAs exert their actions through interactions with chromatin in the regulation of gene expression [17–20], modulation of epigenetic regulation pre- and post-transcriptionally [21–23], and influences on activities and locations of other functional molecules such as proteins and other RNA species [12, 14, 24–29]. Studies have shown that disruption of lncRNA action occurs in certain diseases including cancer [30–34].

In search of the Gene Expression Omnibus (GEO) database from the NCBI website, we found that a novel long intergenic non-coding RNA, *LINC00472* [35] (Supplementary Figure S1), frequently appeared on top of the gene transcript lists that are associated with tumor grade or disease death in several breast cancer microarray datasets. We searched the literature and found no information on this lincRNA with regard to its biological functions and associations with cancer or other diseases. To determine the clinical relevance of *LINC00472* in breast cancer and to assess its biologic effects on breast cancer cells, we measured *LINC00472* expression in more than 300 breast tumor samples to analyze its association with clinical and pathological features of breast cancer, conducted a meta-analysis on more than 2 dozens independent clinical studies to confirm the findings of our clinical study, and transfected a *LINC00472* expression vector into breast cancer cells to assess the lincRNA's effects on cell growth and migration. In this report, we describe the findings of *LINC00472* in our clinical study, meta-analysis and *in vitro* experiments.

## RESULTS

### Clinical study

**Figure 1:**
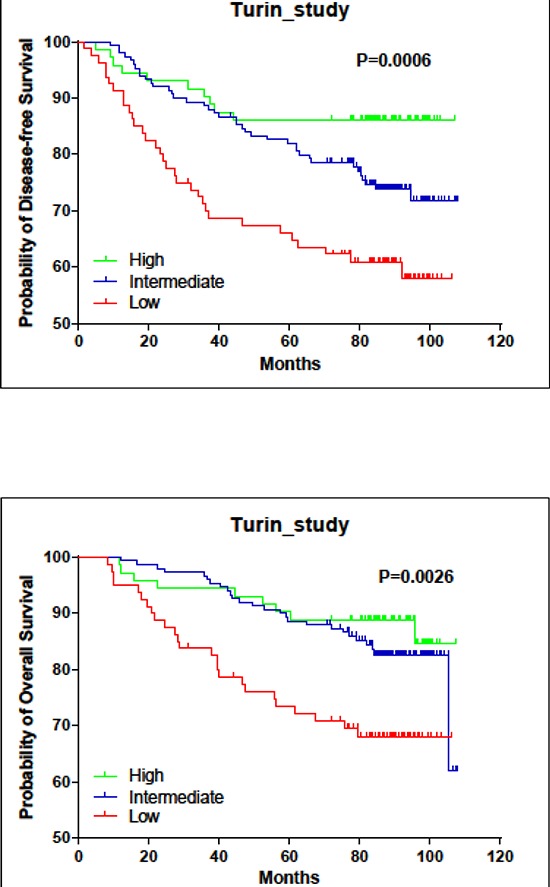
Associations of *LINC00472* expression with patient survival in turin study **A.** Kaplan-Meier estimates for disease-free survival by *LINC00472* expression. **B.** Kaplan-Meier estimates for overall survival by *LINC00472* expression.

In the clinical study of 348 tumor samples, high *LINC00472* expression occurred more often in patients with smaller tumors (*p* < 0.0001), lower tumor grades (*p* < 0.0001), and earlier stage disease (*p* = 0.007) (Table 1). Also, patients with positive hormone receptors had higher *LINC00472* expression compared to those with negative receptor status (*p* < 0.0001 for ER or PR) (Table 1). Furthermore, patients with high expression had better responses to adjuvant chemotherapy (*p* = 0.021) and hormonal therapy (*p* = 0.003) than those with low expression (Table 1). Finally, survival analysis suggested that patients with high expression had better disease-free (*p* < 0.001) and overall survival (*p* = 0.005) compared to those with low expression (Figures 1A, 1B, and Table 2). Risk reduction in relapse was also observed when disease stage, tumor grade, receptor status and other clinical features of the patients were adjusted in analysis (*p* = 0.043) (Table 2).

**Table 1:** Associations of *LINC00472* with Clinicopathological Factors in Turin Study

Patient Features	Total No. (%)	Low LincRNA No. (%)	Mid LincRNA No. (%)	High LincRNA No. (%)	*P* value
**Disease Stage**	346				0.007
Stage 1	128 (36.99)	18 (14.06)	71 (55.47)	39 (30.47)	
Stage 2	183 (52.89)	58 (16.76)	83 (23.99)	42 (22.95)	
Stage 3 & 4	35 (10.12)	10 (28.57)	19 (54.29)	6 (17.14)	
**Tumor Grade**	343				< 0.0001
Grade 1	57 (16.62)	3 (5.26)	27 (47.37)	27 (47.37)	
Grade 2	141 (41.11)	22 (15.60)	80 (56.74)	39 (27.66)	
Grade 3	145 (42.27)	62 (42.76)	62 (42.76)	21 (14.48)	
**Histology Type**	347				0.043
Ductal	219 (63.11)	65 (29.68)	103 (47.03)	51 (23.29)	
Lobular	56 (16.14)	6 (10.71)	29 (51.79)	21 (37.50)	
Mix	35 (10.09)	6 (17.14)	21 (60.00)	8 (22.86)	
Others	37 (10.66)	10 (27.03)	20 (54.05)	7 (18.92)	
**ER Status**	342				< 0.0001
Positive	222 (64.91)	32 (14.41)	118 (53.15)	72 (32.43)	
Negative	120 (35.09)	54 (45.00)	51 (42.50)	15 (12.50)	
**PR Status**	341				< 0.0001
Positive	178 (52.20)	26 (14.61)	98 (55.06)	54 (30.34)	
Negative	163 (47.80)	59 (36.20)	71 (43.56)	33 (20.25)	
**Nodal Status**	347				0.475
Positive	160 (46.11)	45 (28.13)	77 (48.13)	38 (23.75)	
Negative	187 (53.89)	42 (22.46)	96 (51.34)	49 (26.20)	
**Tumor Size**	346				< 0.0001
T1	201 (58.09)	31 (15.42)	106 (52.74)	64 (31.84)	
T2	120 (34.68)	46 (38.33)	54 (45.00)	20 (16.67)	
T3/T4	25 (7.23)	9 (36.00)	13 (52.00)	3 (12.00)	
**Adjuvant Endo-therapy**	173				0.003
Complete Response	135 (78.03)	17 (12.59)	71 (52.59)	47 (34.81)	
No complete response	38 (21.97)	13 (34.21)	19 (50)	6 (15.79)	
**Adjuvant Chemo-therapy**	219				0.021
Complete Response	158 (72.15)	43 (27.22)	75 (47.47)	40 (25.32)	
No complete response	61 (27.85)	25 (40.98)	30 (49.18)	6 (9.84)	

**Table 2:** Associations of *LINC00472* with Breast Cancer Survival in Turin Study

Unadjusted Cox Regression Model						
LincRNA	HR^1^ for relapse	95% CI^2^	*p* value	HR for death	95% CI	*p* value
** Low**	1			1		
** Mid**	0.55	0.34–0.88	0.012	0.47	0.27–0.81	0.006
** High**	0.28	0.14–0.58	< 0.001	0.34	0.16–0.72	0.005
** Continuous**	0.54	0.39–0.74	<0.001	0.55	0.38–0.80	0.002
Adjusted Cox Regression Model*						
**LincRNA**	**HR for relapse**	**95% CI**	***p* value**	**HR for death**	**95% CI**	***p* value**
** Low**	1			1		
** Mid**	0.82	0.48–1.39	0.45	0.62	0.33–1.16	0.134
** High**	0.44	0.20–0.97	0.043	0.51	0.21–1.22	0.129
** Continuous**	0.70	0.48–1.00	0.050	0.69	0.45–1.07	0.098

* Adjusted for age, stage, grade, histology, ER, PR, nodal status and adjuvant treatment.

1 HR: Hazards Ratio

2 CI: Confidence Interval

### Meta-analysis of GEO data

**Figure 2:**
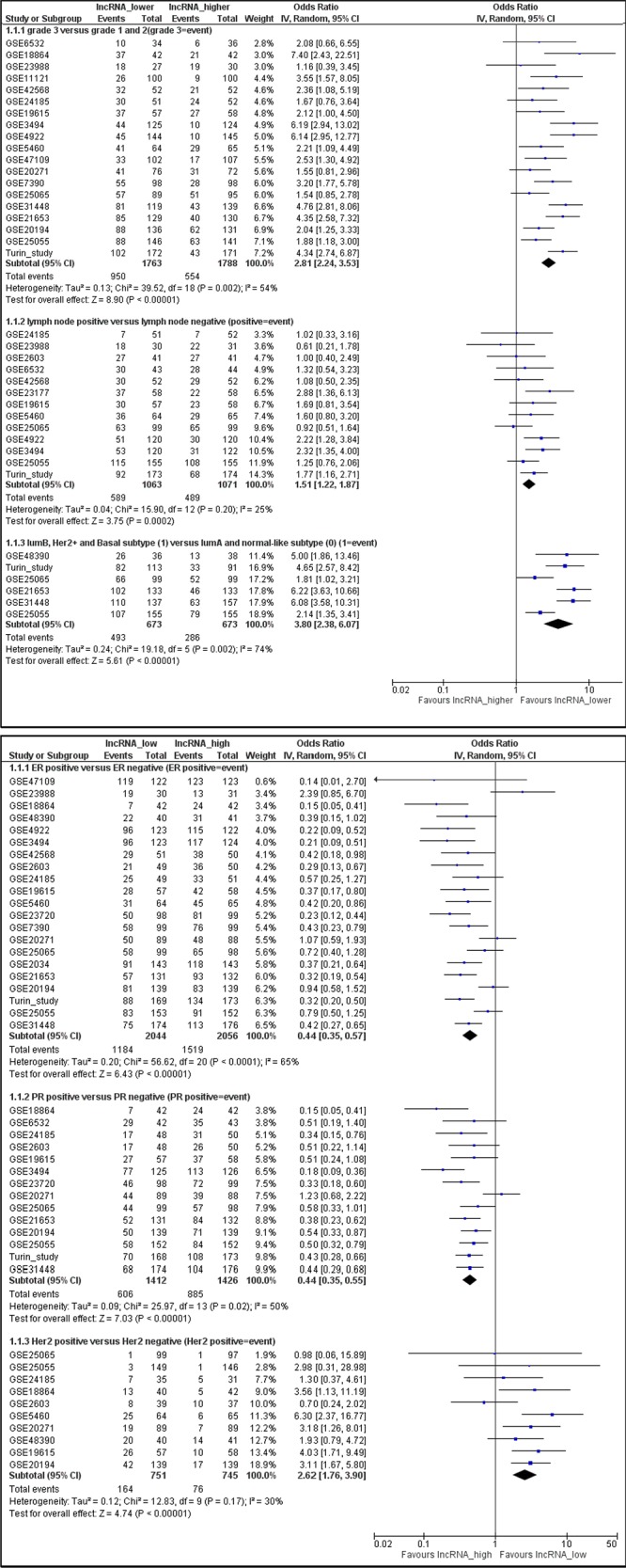
Meta-analysis of associations between *LINC00472* expression and clinicopathological features of breast cancer Summarized odds ratios were estimated using the random-effect model, and the odds ratio in each study was weighted with the variance of probe values (inverse-variance weighted method). **A.** Lower *LINC00472* expression associated with high tumor grade (OR = 2.81; 95% CI: 2.24–3.53), positive lymph node (OR = 1.51; 95% CI: 1.22–1.87), or molecular subtypes of luminal B, Her2 positive and basal-like (OR = 3.80; 95% CI: 2.38–6.07). **B.** Higher *LINC00472* expression associated with ER positive tumors (OR = 0.44; 95% CI: 0.35–0.57), PR positive tumors (OR = 0.44; 95% CI: 0.35–0.55), or Her2 negative tumors (OR = 2.62; 95% CI: 1.76–3.90).

**Figure 3:**
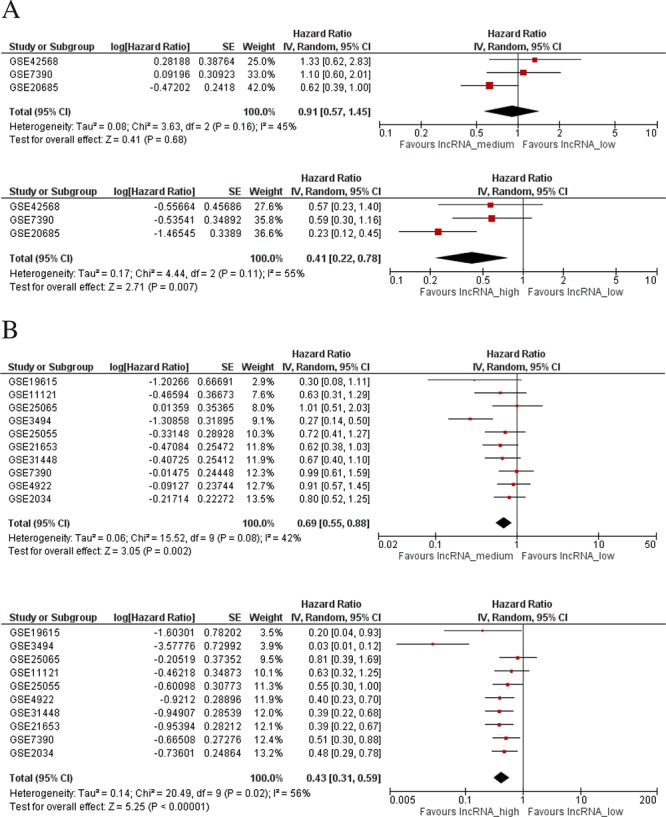
Meta-analysis of associations between LINC00472 expression and patient survival **A.** Overall survival: HR = 0.91 (95% CI: 0.57–1.45) between mid and low *LINC00472* expression (Upper Figure), and HR = 0.41 (95% CI: 0.22–0.78) between high and low *LINC00472* expression (Lower Figure). **B.** Disease-free Survival: HR = 0.69 (95% CI: 0.55–0.88) between mid and low *LINC00472* expression (Upper Figure), and HR = 0.43 (95% CI: 0.31–0.59) between high and low *LINC00472* expression (Lower Figure).

Twenty-seven datasets containing clinicopathologic information were identified in the GEO database (Supplementary Table S1). In a meta-analysis, we found higher *LINC00472* expression to be associated with well-differentiated tumors (low grades) and less aggressive disease (positive ER or PR, negative lymph nodes, luminal A and normal-like molecular subtypes) (Figures 2A, 2B). In GEO, 4 datasets compared gene expression between normal breast tissues and tumors. The comparison showed higher *LINC00472* in normal than in tumor tissues (Supplementary Figure S2). Furthermore, 15 datasets in GEO contained information on various survival outcomes, of which 12 included more than 100 patients individually. We performed survival analysis on these 12 datasets after *LINC00472* expression was grouped into 3 categories similar to those of our own study. Of the 12 studies, 9 showed significant individual associations between high *LINC00472* and favorable survival outcomes (Supplementary Figure S3). Meta-analysis showed both overall survival (Figure 3A) and disease-free survival (Figure 3B) were significantly improved in patients with high *LINC00472* compared to those with low expression. Overall, the results of these analyses were in agreement with our own study.

### *In vitro* experiments

**Figure 4:**
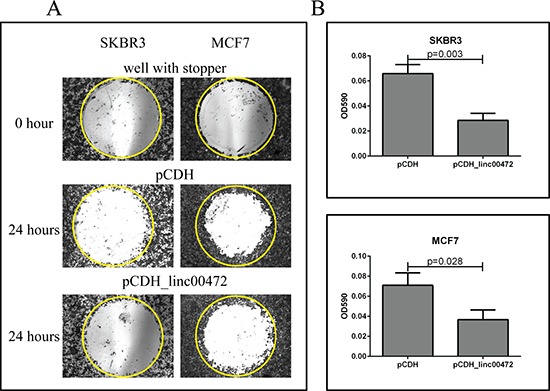
Effect of *LINC00472* expression on breast cancer cell proliferation **A.** GFP fluorescence images in MCF7 and SKBR3 cells transfected with pCDH or pCDH_*LINC00472* vectors. **B.** RT-qPCR results of *LINC00472* expression in MCF7 and SKBR3 cells transfected with pCDH or pCDH_*LINC00472* or mock transfected. **C.** Cell growth inhibition by *LINC00472* in MCF7 cells. Twenty hours after seeding in 96-well plates, cells were transiently transfected with pCDH or pCDH_*LINC00472* vectors, and kept in culture for up to 96 hours. Absorbance at 450nm of each well, which was directly proportional to the number of living cells in the well, was measured by SpectraMax M3 Multimode Plate Reader. The y axis showed the relative absorbance of corresponding wells from different days compared to that from day 0. Error bars represent SEM, *n* = 15. *P* values were determined by the Mann-Whitney *U* test. **D.** Inhibition of cell growth by *LINC00472* in SKBR3 cells. The experiment and analysis are same as to those in C.

**Figure 5:**
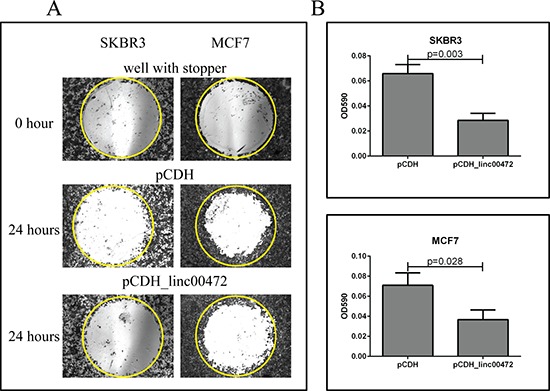
Effect of *LINC00472* expression on breast cancer cell migration **A.** Microscopic views of cell migration before (0 hour) and after (24 hour) removal of stopper in SKBR3 and MCF7 cells transfected with pCDH or pCDH_*LINC00472*. Cells transfected with pCDH or pCDH_*LINC00472* vectors formed monolayers in Oris 96-well plate and started to migrate to the exposed area after removing the stoppers in the well. Twenty-four hours later, the cells were fixed and stained with 0.1% crystal violet stain. The photomicrograph of the entire ‘wound’ area was taken under the IX71 inverted microscope with 4X objective lens. The representative wells were presented and the yellow circles indicated the areas previously occupied by stoppers. **B.** Measurements of absorbance after removal of stopper in SKBR3 and MCF7 cells transfected with pCDH or pCDH_*LINC00472*. With the detection mask, the absorbance at 590 nm wavelength of each well, which was directly proportional to the number of cells that migrated into the ‘wound’ area, was measured. The bar charts showed the average absorbance (y axis) from the wells with different cells after subtracting the background, the absorbance of the reference wells. Error bars represent SEM, *n* = 8. *P* values were determined by the Mann-Whitney *U* test.

To examine the effect of *LINC00472* on breast cancer cells, we analyzed *LINC00472* expression in MCF7 and SKBR3, and found that the expression was low in these cell lines. We created an expression vector, pCDH_*LINC00472* (Supplementary Figure S4), and transfected the vector into breast cancer cells to increase *LINC00472* expression. The transfection was successful based on the GFP signal and RT-qPCR analysis (Figure 4). Cell proliferation assays showed that *LINC00472* expression significantly inhibited tumor cell growth (Figure 4). Furthermore, in the cell migration experiments, we found that both MCF7 and SKBR3 cell lines exhibited reduced migration after being transfected with *LINC00472* expressing vectors (Figure 5). These *in vitro* experiments suggest that increased *LINC00472* expression may suppress tumor cell proliferation and migration, which is consistent with the findings of our clinical studies.

## DISCUSSION

Our clinical study showed that *LINC00472* expression was significantly different by tumor grade, tumor size, disease stage, receptor status and molecular subtype. The expression was also associated with treatment response and survival outcomes, suggesting potential prognostic and predictive implications in clinical management of breast cancer. More importantly, our findings are very consistent across multiple datasets. This consistency is established on the basis of more than 20 independent studies which collectively included thousands of patients. The consistent finding was also based on two different laboratory methods, qPCR and microarray chips. Furthermore, our *in vitro* experiments on breast cancer cell lines supported the findings of our clinical studies. The overall consistency among multiple clinical studies and between *in vivo* and *in vitro* systems underscores the potential significance of *LINC00472*'s involvement in breast cancer, especially in tumor progression.

The gene encoding *LINC00472* is located on chromosome 6q13, and the verified transcript has 2, 933 bp (NR_026807.1). The earlier annotation also contained a predicted transcript (XR_241853.1) that is longer than 6,000 bp. We had designed two pairs of RT-qPCR primers to analyze both transcripts, and found that they were highly correlated (*r* = 0.76, *p* < 0.0001). Therefore, we chose the one which covers both transcripts for our measurement of expression in the study. Downstream from the *LINC00472* gene, there are two microRNA genes, miR30c and miR30a (Supplementary Figure S1), which have been reported to have possible effects on breast cancer invasion, metastasis and patient response to chemotherapy [36–42]. Although *LINC00472* has not yet been functionally characterized for cellular activities or molecular processes, the results from multiple independent datasets and different platforms suggest that this lincRNA may behave like a tumor suppressor. Our *in vitro* experiments also indicates that *LINC00472* can suppress cell proliferation and migration. We notice that the deletion of this gene is rare in the TCGA data [43, 44], and therefore the low expression of *LINC00472* in breast cancer is likely due to the down-regulation of expression. The biological mechanisms that control the expression of *LINC00472* are still unknown.

Very recently a new clone (RP1–288M22) on chromosome 6q12–13 was submitted to NCBI. This clone expands the *LINC00472* transcript to more than 9,000 bp (NR_026807.2) with three additional transcript variants of similar size. Our RT-qPCR primers were able to detect all four transcripts according to the NCBI Blast results (Supplementary Figure S5). Concerning the probes in the microarray chip from which the GEO data were generated, one probe (235771_at) represents the new clone. We compared the expression of this probe with the one used in our study (220324_at) in 15 datasets where data were available, and found a strong correlation of expression between these probes (Supplementary Figure S6), suggesting that our findings of *LINC00472* expression in association with breast cancer based on 220324_at be applicable to the recent submission or renewal of *LINC00472*.

Long non-coding RNAs are suspected to exert their functions through interacting with ribonucleoprotein (RNP), but experimentally elucidating the actions of RNP complexes is difficult and time-consuming. To date, only a few RNP complexes have been characterized experimentally. Thus, computational prediction may be a valuable alternative to help predict the possible actions of *LINC00472*. We used a sequence-based method, *cat*RAPID *omics*, to search for the binding proteins of *LINC00472*. Since *cat*RAPID *omics* uses an algorithm that combines multiple features in addition to primary structure, its prediction is considered to be relatively reliable. Importantly this method has been used previously for testing several long non-coding RNAs, such as Xist, the long non-coding RNA X-inactive-specific transcript, and the results of prediction have been consistent with experimental findings. The *LINC00472*-binding proteome returned from the *cat*RAPID *omics* analysis contained a total of 579 proteins, from which 127 showed high interaction propensities (Interaction Strength > 50% and Discriminative Power > 75%, Z-score > 1.0) (Supplementary Figure S7). We uploaded these proteins onto the Ingenuity Pathway Analysis (IPA) System for a core analysis using the Ingenuity Knowledge Base as a reference set. The IPA analysis suggested that proteins interacting with *LINC00472* may involve several diseases and disorders, of which cancer is on top of the list (Supplementary Figure S7). IPA analysis also indicated that these proteins may be functionally involved in RNA post-transcriptional modification and protein synthesis and associated with 3 signaling networks where multiple biomarkers are known for breast cancer diagnosis, prognosis and treatment prediction (Supplementary Figures S8A–S8C). Knowing the potential RNA binding-proteins may allow us not only to predict the possible targets of the lincRNA, but also to design additional experiments to assess its biologic functions.

Microarray technologies have substantially enhanced the search of biomarkers for cancer prognosis, but long non-coding RNAs in data generated by microarray analysis have not been well interrogated. Using GEO2R, an interactive web tool in GEO, we analyzed the relationships of *LINC00472* expression and breast cancer characteristics in multiple GEO datasets generated from the most recent microarray chips, the Affymetrix Human Genome U133 plus 2.0 array and the U133A array. Our finding of a consistent association between *LINC00472* expression and breast cancer survival among various datasets supports the validity of this association and indicates such public databases invaluable to biomedical research. The consistency in the associations with clinicopathologic variables further strengthens the finding of our study.

Overall, our investigation was based on multiple studies from diverse patient populations, and involved a large number of patients. We also demonstrated the results with different methods of lncRNA analysis (microarray and qPCR). These differences in patient populations and lab techniques lend strong support to our findings of *LINC00472* in breast cancer. However, at present, very little is known about the exact functions of this long non-coding RNA. Therefore, we have not discussed the possible biologic mechanisms that may explain why *LINC00472* behaves like a tumor suppressor. Whether our findings reflect direct effects of *LINC00472* on breast cancer or indirect actions via other molecules is unclear. More work is needed to elucidate the function of *LINC00472* and its role in breast tumorigenesis.

## MATERIALS AND METHODS

The role of *LINC00472* in breast cancer was investigated through a) a clinical study in which we analyzed *LINC00472* expression in 348 tumors using RT-qPCR, b) meta-analysis of microarray data deposited in the GEO database, and c) manipulation of *LINC00472* expression in breast cancer cell lines.

### Clinical study (Turin_Study)

#### Breast cancer patients

A clinical study of breast cancer was conducted in the University Hospital at University of Turin, in Italy, between January 1998 and July 1999. The study was approved by the university's ethics review committee. During the study, 348 breast cancer patients were enrolled and provided informed consent. All study patients underwent surgical resection for breast cancer. Average age at surgery was 57 years (range: 23–84 years). Of the patients enrolled, 302 had follow-up information available through February 2007. Median follow-up was 86 months (range: 8–108 months). During follow-up, 81 patients developed recurrent or metastatic disease, and 55 of them died from the disease. A total of 60 died by the end of follow-up. Among the patients enrolled, 36.4% had Stage I disease (TMN), 53.4% had Stage II, and 10.3% had Stage III or IV. Patients diagnosed with Grade 1 (well differentiated) tumors comprised 16.6%, Grade 2 41.1%, and Grade 3 42.3%. Ductal carcinoma accounted for the majority of cases (63.1%), followed by lobular carcinoma (16.1%), other specific types (10.7%), and mixed histologic types (10.1%). Two hundred one patients (58.1%) had tumors smaller than 2 cm, 120 (34.7%) had tumors between 2 and 5 cm, and 25 (7.2%) had tumors greater than 5 cm. One hundred sixty patients (46.8%) had lymph node-positive tumors. Sixty-five percent of the patients had ER positive tumors, and 52.2% had PR positive tumors (at 10% cutoff for receptor positivity). Of the 348 patients, 303 received adjuvant therapy after surgery. Of those treated, 119 (34.2%) had chemotherapy, 77 (22.1%) had hormonal therapy, and 107 (30.8%) received both. The chemotherapy protocols administered included CMF (cyclophosfamide, methotrexate, 5 fluorouracil; 600/60/600 mg/mq every three weeks for six cycles), CEF (cyclophosfamide, epirubicin, 5 fluorouracil; 600/90/600 mg/mq every three weeks for six cycles), EPI-TAX (epirubicin-paclitaxel; 90/175 mg/mq every three weeks for six cycles), EPI-VNB (epirubicin-vinorelbine; 90/30 mg/mq every three weeks for six cycles), DTX-EPI-VNB (doxetaxel-epirubicin-vinorelbine; 75/90/30 mg/mq every three weeks for six cycles), and TXT-EPI-VNB (paclitaxel-epirubicin-vinorelbine; 175/90/30 mg/mq every three weeks for cycles). Tamoxifen was the only agent used for endocrine therapy at that time. The dose was one 20 mg tablet per day for five years or until disease progression or intolerable toxicity.

#### Analysis of *LINC00472* expression

Fresh tumor samples collected from patients during surgery were snap-frozen in liquid nitrogen immediately after resection and stored at −80°C until analysis. All tissue samples were examined by pathologists to confirm at least 80% tumor content. The tissue samples (~30 mg each) were homogenized with ceramic beads in PowerLyzer (MO BIO), and processed to extract total RNA using Allprep DNA/RNA Kit (Qiagen). The RNA samples were treated with RNase-free DNase and quantified using a spectrophotometer.

PCR primers were designed using the sequence NR_026807.1 (Supplementary Figure S1) and the synthesis was done by IDT (San Diego, CA). Total RNA (1 μg) was reverse transcribed using the cDNA Reverse Transcription Kit (LifeTech); RT-qPCR was performed in triplicate using SYBR Select Master Mix (LifeTech). In PCR reaction (10 μl), cDNA template (0.5 μl) was mixed with 200 nM primers and 5 μl SYBR PCR master mix. PCR conditions were incubation at 50°C for 2 min to activate UDG, 95°C for 2 min to activate Taq polymerase, and 40 cycles of 95°C for 15 s and 60°C for 1 min. *LINC00472* measurement was normalized to GAPDH using the formula described in Statistical Analysis.

### Meta-analysis of GEO data

Gene expression data generated from the Affymetrix Human Genome U133 plus 2.0 array and U133A array were selected from the GEO database. These datasets include 4, 628 breast cancer samples (Supplementary Table S1) and 193 normal breast tissues (Supplementary Figure S2). Expression data from probe 220324_at (which targets *LINC00472*) were used for meta-analysis, in which normalized expression was dichotomized using study-specific median expression as cutoff to define “*LINC00472*_higher” at or above median versus “*LINC00472*_lower” below median. Clinical and pathologic variables were also dichotomized. For each variable, summary odds ratios and their 95% confidence intervals were estimated using the inverse variance weighted method. Because the meta-analysis involved expression data assessed by different methods, we used the random-effects model. Forest plots were used to present the results. The meta-analysis was conducted with the use of Review Manager (Revman Version 5.3, Copenhagen, Denmark). Cochran χ^2^ test and I^2^ statistic were used to assess the heterogeneity among the studies involved. For datasets with more than 100 patients, Kaplan-Meier survival analyses were performed on individual studies (Supplementary Figure S3).

### *In vitro* experiments

#### Preparation of *LINC00472* construct

A *LINC00472* transcript (2933 bp, NR_026807.1) was assembled using the EST clones, EHS1001–207275390, EHS1001–207498495, EHS1001–207533792, EHS1001–207590772, EHS1001–210281579, and EHS1001–211231922 (Thermo Scientific Open Biosystems). Restriction digestion sites for NheI and BamHI were incorporated into each site of the *LINC00472* transcript for cloning and verification. The entire sequence was inserted into a lentiviral expression vector, pCDH-EF1-MCS-pA-PGK-copGFP-T2A-Puro (System Biosciences). The construction was done by Science Exchange (Supplementary Figure S4). The clone authenticity was confirmed by restriction enzyme digestion (Supplementary Figure S4) and sequencing analysis.

#### Cell culture experiments

Breast cancer cell lines, SKBR3 and MCF-7, were a kind gift from Jun Panee (JABSOM, University of Hawaii) and were grown in DMEM medium containing 10% FBS and 100 units/ml of penicillin/streptomycin (Pen/Strep). We transfected the cells with the *LINC00472* vector (pCDH_*LINC00472*), along with mock transfection and transfection of a control vector (pCDH). After 24 h incubation, cells were examined for GFP fluorescence; the evaluation was repeated in 48 h. After that, total RNA was extracted from the cells, and reverse-transcribed for qPCR analysis of *LINC00472* expression which was normalized to GAPDH and compared between cells with different transfection conditions.

#### Cell proliferation assay

Twenty hours after seeding in 96-well plates, cells were transiently transfected with plasmids or mock-transfected, and kept growing for up to 96 h. At the end of each incubation period (24, 48, 72 and 96 h), numbers of viable cells were assessed by Cell Counting Kit-8 (Sigma-Aldrich).

#### Cell migration assay

Cell migration was assessed with the modified Oris Cell Migration Assay (AMSBIO). After 24 hours of transfection with plasmids pCDH or pCDH_*LINC00472*, equal amount of cells were plated into the Oris 96-well plate which was incubated overnight with stoppers to permit cell attachment. Stoppers were removed from wells except the reference wells in which the stoppers were remained in place until results were read as pre-migration controls. Media was removed and wells were gently washed with sterile PBS. After that, fresh culture media was added to each well, and the plate was incubated for another 24 hours to allow for cell migration. The cultured cells were fixed and stained with 0.1% crystal violet stain. The photomicrograph of the entire wound area was taken using the IX71 inverted microscope with 4X objective lens. Using a detection mask, the absorbance at 590 nm wavelength in each well, which was directly proportional to the number of cells that migrated into the ‘wound’ area, was measured by a spectrophotometer (SpectraMax M3 Multimode Plate Reader).

### Statistical analysis

*LINC00472* expression was calculated as an expression index (EI), using the formula 1,000 × 2^(–ΔCt)^, where ΔCt = Ct (*LINC00472*) – Ct (GAPDH). For data analysis, EI was analyzed as a categorical variable with 3 ordinal levels, low (< 25 percentile), Intermediate (25–75 percentile, labeled as “Mid” in Table 1), and high expression (> 75 percentile). Associations of *LINC00472* with clinical, pathologic and treatment variables and survival outcomes were determined using the Chi-square test or Cox proportional hazards regression model, as appropriate. Kaplan-Meier survival curves were constructed to show survival differences according to *LINC00472* expression. The survival time for either overall or disease-free was calculated as the time from surgery until the occurrence of death and relapse, respectively. The Mann-Whitney *U* test was used for comparing differences in cell counts and migration. Spearman correlation coefficients were calculated for correlation analysis. All statistical tests were two-sided, and a *p* value less than 0.05 was considered as statistical significance.

## SUPPLEMENTAL FIGURES AND TABLES



## References

[1] Ferlay JSI, Ervik M, Dikshit R, Eser S, Mathers C, Rebelo M, Parkin DM, Forman D, Bray F (2013). GLOBOCAN 2012 v1.0, Cancer Incidence and Mortality Worldwide: IARC CancerBase No. 11 [Internet]. International Agency for Research on Cancer.

[2] Bertos NR, Park M (2011). Breast cancer - one term, many entities?. The Journal of clinical investigation.

[3] Burrell RA, McGranahan N, Bartek J, Swanton C (2013). The causes and consequences of genetic heterogeneity in cancer evolution. Nature.

[4] Roylance R, Endesfelder D, Gorman P, Burrell RA, Sander J, Tomlinson I, Hanby AM, Speirs V, Richardson AL, Birkbak NJ, Eklund AC, Downward J, Kschischo M, Szallasi Z, Swanton C (2011). Relationship of extreme chromosomal instability with long-term survival in a retrospective analysis of primary breast cancer. Cancer epidemiology, biomarkers & prevention: a publication of the American Association for Cancer Research, cosponsored by the American Society of Preventive Oncology.

[5] Stephens PJ, Tarpey PS, Davies H, Van Loo P, Greenman C, Wedge DC, Nik-Zainal S, Martin S, Varela I, Bignell GR, Yates LR, Papaemmanuil E, Beare D, Butler A, Cheverton A, Gamble J (2012). The landscape of cancer genes and mutational processes in breast cancer. Nature.

[6] Ding L, Ellis MJ, Li S, Larson DE, Chen K, Wallis JW, Harris CC, McLellan MD, Fulton RS, Fulton LL, Abbott RM, Hoog J, Dooling DJ, Koboldt DC, Schmidt H, Kalicki J (2010). Genome remodelling in a basal-like breast cancer metastasis and xenograft. Nature.

[7] Nik-Zainal S, Van Loo P, Wedge DC, Alexandrov LB, Greenman CD, Lau KW, Raine K, Jones D, Marshall J, Ramakrishna M, Shlien A, Cooke SL, Hinton J, Menzies A, Stebbings LA, Leroy C (2012). The life history of 21 breast cancers. Cell.

[8] International Human Genome Sequencing C (2004). Finishing the euchromatic sequence of the human genome. Nature.

[9] Kapranov P, Willingham AT, Gingeras TR (2007). Genome-wide transcription and the implications for genomic organization. Nature reviews Genetics.

[10] Huarte M, Rinn JL (2010). Large non-coding RNAs: missing links in cancer?. Human molecular genetics.

[11] Ji P, Diederichs S, Wang W, Boing S, Metzger R, Schneider PM, Tidow N, Brandt B, Buerger H, Bulk E, Thomas M, Berdel WE, Serve H, Muller-Tidow C (2003). MALAT-1, a novel noncoding RNA, and thymosin beta4 predict metastasis and survival in early-stage non-small cell lung cancer. Oncogene.

[12] Huarte M, Guttman M, Feldser D, Garber M, Koziol MJ, Kenzelmann-Broz D, Khalil AM, Zuk O, Amit I, Rabani M, Attardi LD, Regev A, Lander ES, Jacks T, Rinn JL (2010). A large intergenic noncoding RNA induced by p53 mediates global gene repression in the p53 response. Cell.

[13] Mourtada-Maarabouni M, Pickard MR, Hedge VL, Farzaneh F, Williams GT (2009). GAS5, a non-protein-coding RNA, controls apoptosis and is downregulated in breast cancer. Oncogene.

[14] Yu W, Gius D, Onyango P, Muldoon-Jacobs K, Karp J, Feinberg AP, Cui H (2008). Epigenetic silencing of tumour suppressor gene p15 by its antisense RNA. Nature.

[15] Gupta RA, Shah N, Wang KC, Kim J, Horlings HM, Wong DJ, Tsai MC, Hung T, Argani P, Rinn JL, Wang Y, Brzoska P, Kong B, Li R, West RB, van de Vijver MJ (2010). Long non-coding RNA HOTAIR reprograms chromatin state to promote cancer metastasis. Nature.

[16] Prensner JR, Iyer MK, Balbin OA, Dhanasekaran SM, Cao Q, Brenner JC, Laxman B, Asangani IA, Grasso CS, Kominsky HD, Cao X, Jing X, Wang X, Siddiqui J, Wei JT, Robinson D (2011). Transcriptome sequencing across a prostate cancer cohort identifies PCAT-1, an unannotated lincRNA implicated in disease progression. Nature biotechnology.

[17] Wang X, Arai S, Song X, Reichart D, Du K, Pascual G, Tempst P, Rosenfeld MG, Glass CK, Kurokawa R (2008). Induced ncRNAs allosterically modify RNA-binding proteins in cis to inhibit transcription. Nature.

[18] Feng J, Bi C, Clark BS, Mady R, Shah P, Kohtz JD (2006). The Evf-2 noncoding RNA is transcribed from the Dlx-5/6 ultraconserved region and functions as a Dlx-2 transcriptional coactivator. Genes & development.

[19] Martianov I, Ramadass A, Serra Barros A, Chow N, Akoulitchev A (2007). Repression of the human dihydrofolate reductase gene by a non-coding interfering transcript. Nature.

[20] Mariner PD, Walters RD, Espinoza CA, Drullinger LF, Wagner SD, Kugel JF, Goodrich JA (2008). Human Alu RNA is a modular transacting repressor of mRNA transcription during heat shock. Molecular cell.

[21] Rinn JL, Kertesz M, Wang JK, Squazzo SL, Xu X, Brugmann SA, Goodnough LH, Helms JA, Farnham PJ, Segal E, Chang HY (2007). Functional demarcation of active and silent chromatin domains in human HOX loci by noncoding RNAs. Cell.

[22] Nagano T, Mitchell JA, Sanz LA, Pauler FM, Ferguson-Smith AC, Feil R, Fraser P (2008). The Air noncoding RNA epigenetically silences transcription by targeting G9a to chromatin. Science.

[23] Pandey RR, Mondal T, Mohammad F, Enroth S, Redrup L, Komorowski J, Nagano T, Mancini-Dinardo D, Kanduri C (2008). Kcnq1ot1 antisense noncoding RNA mediates lineage-specific transcriptional silencing through chromatin-level regulation. Molecular cell.

[24] Yap KL, Li S, Munoz-Cabello AM, Raguz S, Zeng L, Mujtaba S, Gil J, Walsh MJ, Zhou MM (2010). Molecular interplay of the noncoding RNA ANRIL and methylated histone H3 lysine 27 by polycomb CBX7 in transcriptional silencing of INK4a. Molecular cell.

[25] Zhou Y, Zhong Y, Wang Y, Zhang X, Batista DL, Gejman R, Ansell PJ, Zhao J, Weng C, Klibanski A (2007). Activation of p53 by MEG3 non-coding RNA. The Journal of biological chemistry.

[26] Prasanth KV, Prasanth SG, Xuan Z, Hearn S, Freier SM, Bennett CF, Zhang MQ, Spector DL (2005). Regulating gene expression through RNA nuclear retention. Cell.

[27] Bernard D, Prasanth KV, Tripathi V, Colasse S, Nakamura T, Xuan Z, Zhang MQ, Sedel F, Jourdren L, Coulpier F, Triller A, Spector DL, Bessis A (2010). A long nuclear-retained non-coding RNA regulates synaptogenesis by modulating gene expression. The EMBO journal.

[28] Tripathi V, Ellis JD, Shen Z, Song DY, Pan Q, Watt AT, Freier SM, Bennett CF, Sharma A, Bubulya PA, Blencowe BJ, Prasanth SG, Prasanth KV (2010). The nuclear-retained noncoding RNA MALAT1 regulates alternative splicing by modulating SR splicing factor phosphorylation. Molecular cell.

[29] Wilusz JE, Freier SM, Spector DL (2008). 3′ end processing of a long nuclear-retained noncoding RNA yields a tRNA-like cytoplasmic RNA. Cell.

[30] Penny GD, Kay GF, Sheardown SA, Rastan S, Brockdorff N (1996). Requirement for Xist in X chromosome inactivation. Nature.

[31] Fitzpatrick GV, Soloway PD, Higgins MJ (2002). Regional loss of imprinting and growth deficiency in mice with a targeted deletion of KvDMR1. Nature genetics.

[32] Sleutels F, Zwart R, Barlow DP (2002). The non-coding Air RNA is required for silencing autosomal imprinted genes. Nature.

[33] Li L, Liu B, Wapinski OL, Tsai MC, Qu K, Zhang J, Carlson JC, Lin M, Fang F, Gupta RA, Helms JA, Chang HY (2013). Targeted disruption of Hotair leads to homeotic transformation and gene derepression. Cell reports.

[34] Zhang B, Arun G, Mao YS, Lazar Z, Hung G, Bhattacharjee G, Xiao X, Booth CJ, Wu J, Zhang C, Spector DL (2012). The lncRNA Malat1 is dispensable for mouse development but its transcription plays a cis-regulatory role in the adult. Cell reports.

[35] Mungall AJ, Palmer SA, Sims SK, Edwards CA, Ashurst JL, Wilming L, Jones MC, Horton R, Hunt SE, Scott CE, Gilbert JG, Clamp ME, Bethel G, Milne S, Ainscough R, Almeida JP (2003). The DNA sequence and analysis of human chromosome 6. Nature.

[36] Bockhorn J, Yee K, Chang YF, Prat A, Huo D, Nwachukwu C, Dalton R, Huang S, Swanson KE, Perou CM, Olopade OI, Clarke MF, Greene GL, Liu H (2013). MicroRNA-30c targets cytoskeleton genes involved in breast cancer cell invasion. Breast cancer research and treatment.

[37] Zhou H, Xu X, Xun Q, Yu D, Ling J, Guo F, Yan Y, Shi J, Hu Y (2012). microRNA-30c negatively regulates endometrial cancer cells by targeting metastasis-associated gene-1. Oncology reports.

[38] Bockhorn J, Dalton R, Nwachukwu C, Huang S, Prat A, Yee K, Chang YF, Huo D, Wen Y, Swanson KE, Qiu T, Lu J, Park SY, Dolan ME, Perou CM, Olopade OI (2013). MicroRNA-30c inhibits human breast tumour chemotherapy resistance by regulating TWF1 and IL-11. Nature communications.

[39] Rodriguez-Gonzalez FG, Sieuwerts AM, Smid M, Look MP, Meijer-van Gelder ME, de Weerd V, Sleijfer S, Martens JW, Foekens JA (2011). MicroRNA-30c expression level is an independent predictor of clinical benefit of endocrine therapy in advanced estrogen receptor positive breast cancer. Breast cancer research and treatment.

[40] Franzetti GA, Laud-Duval K, Bellanger D, Stern MH, Sastre-Garau X, Delattre O (2013). MiR-30a-5p connects EWS-FLI1 and CD99, two major therapeutic targets in Ewing tumor. Oncogene.

[41] Baraniskin A, Birkenkamp-Demtroder K, Maghnouj A, Zollner H, Munding J, Klein-Scory S, Reinacher-Schick A, Schwarte-Waldhoff I, Schmiegel W, Hahn SA (2012). MiR-30a-5p suppresses tumor growth in colon carcinoma by targeting DTL. Carcinogenesis.

[42] Zhang N, Wang X, Huo Q, Sun M, Cai C, Liu Z, Hu G, Yang Q (2014). MicroRNA-30a suppresses breast tumor growth and metastasis by targeting metadherin. Oncogene.

[43] Gao J, Aksoy BA, Dogrusoz U, Dresdner G, Gross B, Sumer SO, Sun Y, Jacobsen A, Sinha R, Larsson E, Cerami E, Sander C, Schultz N (2013). Integrative analysis of complex cancer genomics and clinical profiles using the cBioPortal. Science signaling.

[44] Cerami E, Gao J, Dogrusoz U, Gross BE, Sumer SO, Aksoy BA, Jacobsen A, Byrne CJ, Heuer ML, Larsson E, Antipin Y, Reva B, Goldberg AP, Sander C, Schultz N (2012). The cBio cancer genomics portal: an open platform for exploring multidimensional cancer genomics data. Cancer discovery.

