# Supplementary material for: Prognostic and predictive values of long non-coding RNA LINC00472 in breast cancer
Source: Oncotarget. 2015 Mar 25;6(11):8579–92. doi: 10.18632/oncotarget.3287 (PMC4496168; doi:10.18632/oncotarget.3287)
Supplement: Supplementary file 1 [file oncotarget-06-8579-s001.pdf]

SUPPLEMENTARY FIGURES AND TABLE

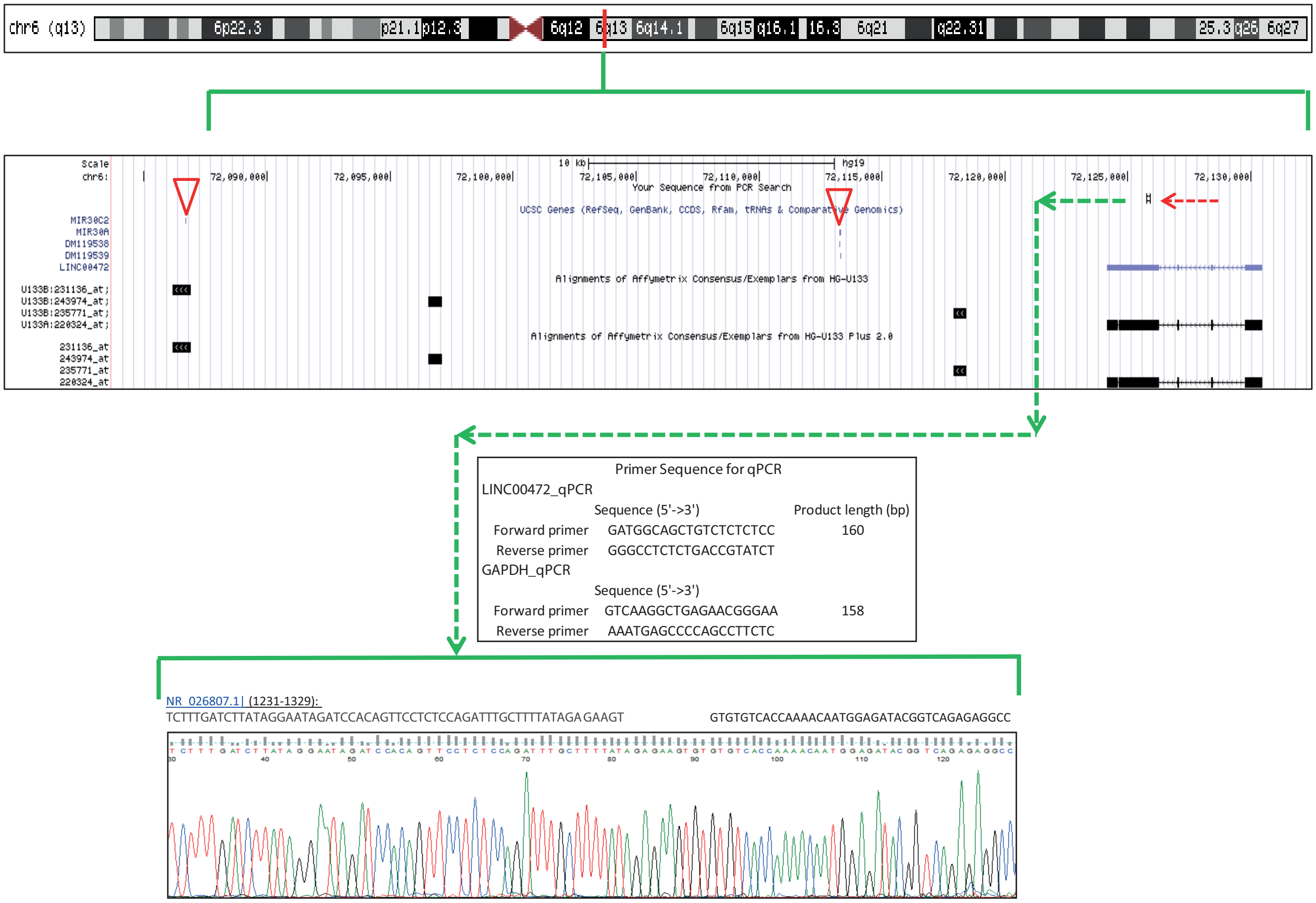

Primer Sequence for qPCR

	Sequence (5'->3')	Product length (bp)
**LINC00472_qPCR**		
Forward primer	GATGGCAGCTGTCTCTCTCC	160
Reverse primer	GGGCCTCTCTGACCGTATCT	
**GAPDH_qPCR**	Sequence (5'->3')	
Forward primer	GTCAAGGCTGAGAACGGGAA	158
Reverse primer	AAATGAGCCCCAGCCTTCTC	

Supplementary Figure S1: *LINC00472* location in chromosome 6 and methods of detection. A schematic diagram shows that *LINC00472* gene is located on chromosome 6q13, complementary strand (red bar). This region is amplified in the following part of the figure. Two microRNA (pointed by red triangles), miR30A and miR30C2, are in the downstream of *LINC00472*. The *LINC00472* transcript (NR_026807.1) includes 4 exons. The red arrow denotes the targeted area of the primers specifically designed for RT-qPCR assay. The black blocks show the targeted area of the probes designed in Affymetrix array HG-U133 and HG-U133 plus 2. The primers' sequences for *LINC00472* and control GAPDH are listed underneath. The RT-qPCR products had been purified and sequenced. The blast result of the PCR product is at the bottom of the figure, showing its match to *LINC00472* transcript NR_026807.1.

A

GEO Series(Ref)	Country	Normal	Breast Cancer	Platform*	Analysis Method
GSE5764[1]	Czech Republic	20	10	GPL570	RMA log 2 expression levels
GSE10780[2]	USA	143	42	GPL570	RMA log 2 expression levels
GSE31192[3]	USA	13	20	GPL570	RMA log 2 expression levels
GSE42568[4]	Ireland	17	104	GPL570	Log2 GC-RMA signal intensity
Total		193	176		

1. Turashvili G, Bouchal J, Baumforth K, et al. Novel markers for differentiation of lobular and ductal invasive breast carcinomas by laser microdissection and microarray analysis. BMC cancer 2007;7:55.
2. Chen DT, Nasir A, Culhane A, et al. Proliferative genes dominate malignancy-risk gene signature in histologically-normal breast tissue. Breast cancer research and treatment 2010;119:335-46.
3. & DMEHJKJOB, & A-CTVFBPS, Horwitz BMJKB. Genomic Signatures of Pregnancy-Associated Breast Cancer Epithelia and Stroma and their Regulation by Estrogens and Progesterone. HORM CANC 2013;4:140-53.
4. Clarke C, Madden SF, Doolan P, et al. Correlating transcriptional networks to breast cancer survival: a large-scale coexpression analysis. Carcinogenesis 2013;34:2300-8.
* GPL570 - Affymetrix Human Genome U133 Plus 2.0 Array

B

Study or Subgroup	lncRNA_low Events	lncRNA_low Total	lncRNA_high Events	lncRNA_high Total	Weight	Odds Ratio IV, Random, 95% CI
GSE31192	13	15	7	16	12.5%	8.36 [1.40, 49.88]
GSE5764	6	15	4	15	15.9%	1.83 [0.39, 8.57]
GSE42568	52	60	52	61	29.0%	1.13 [0.40, 3.14]
GSE10780	29	92	13	93	42.6%	2.83 [1.36, 5.89]
**Total (95% CI)**		**182**		**185**	**100.0%**	**2.31 [1.17, 4.59]**
Total events	100		76			

Heterogeneity: $Tau^2 = 0.15$; $Chi^2 = 4.26$, df = 3 (P = 0.23); $I^2 = 30\%$
Test for overall effect: Z = 2.40 (P = 0.02)

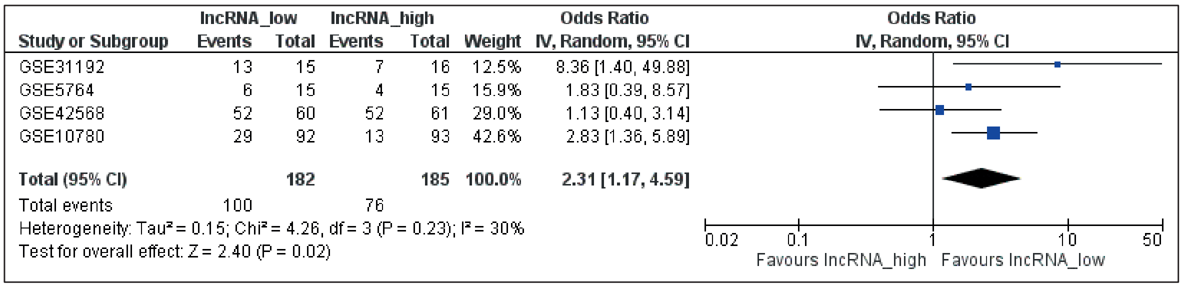

Supplementary Figure S2: *LINC00472* expression in normal breast and tumor tissues. A. Study information on 4 GEO datasets which consist of normal breast and breast cancer samples. **B.** Meta-analysis of the 4 GEO datasets showed higher levels of *LINC00472* expression in normal than in tumor tissues.

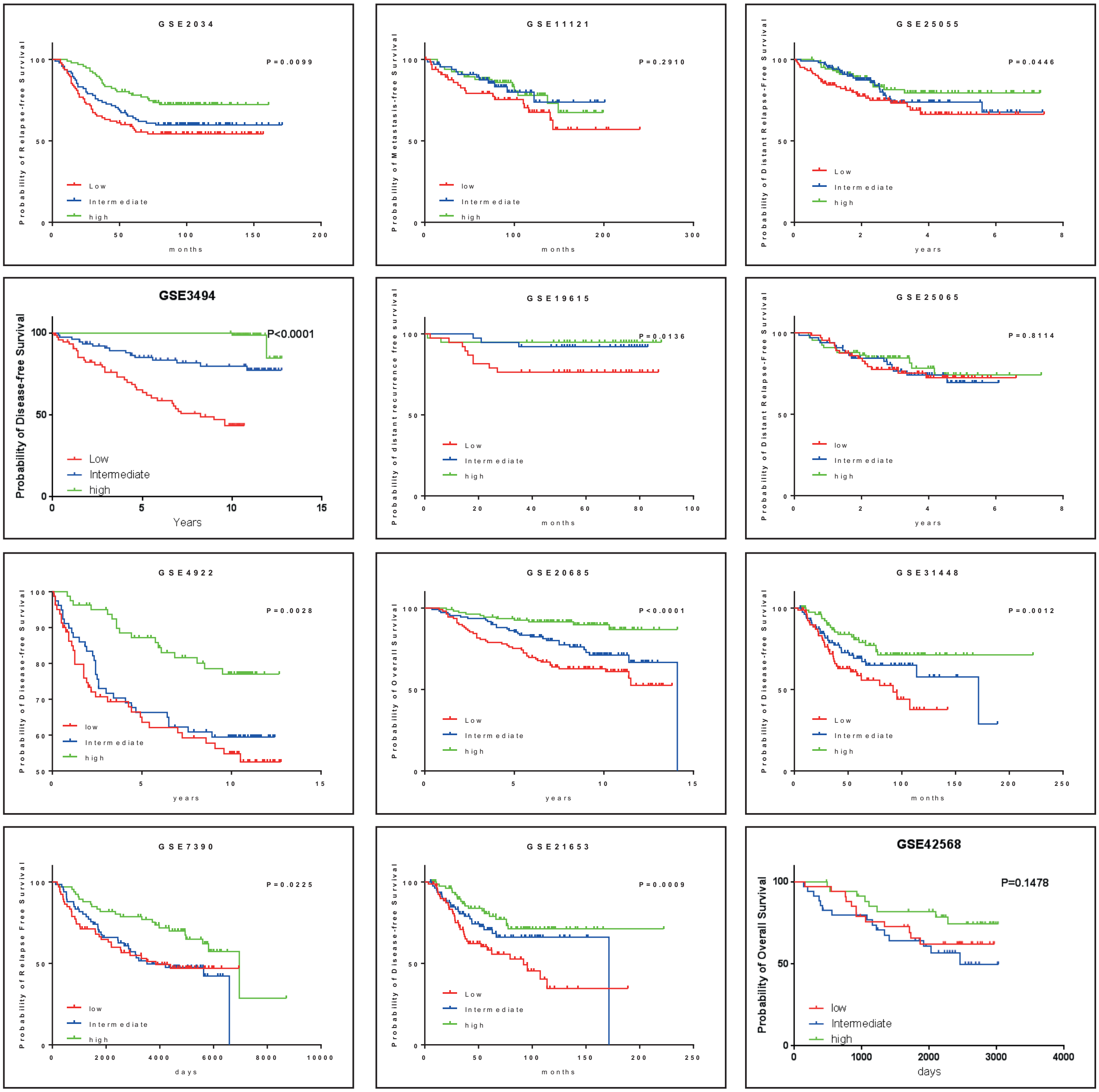

Supplementary Figure S3: Kaplan-meier survival curves by low, medium and high *LINC00472* expression in 12 studies from the GEO database.

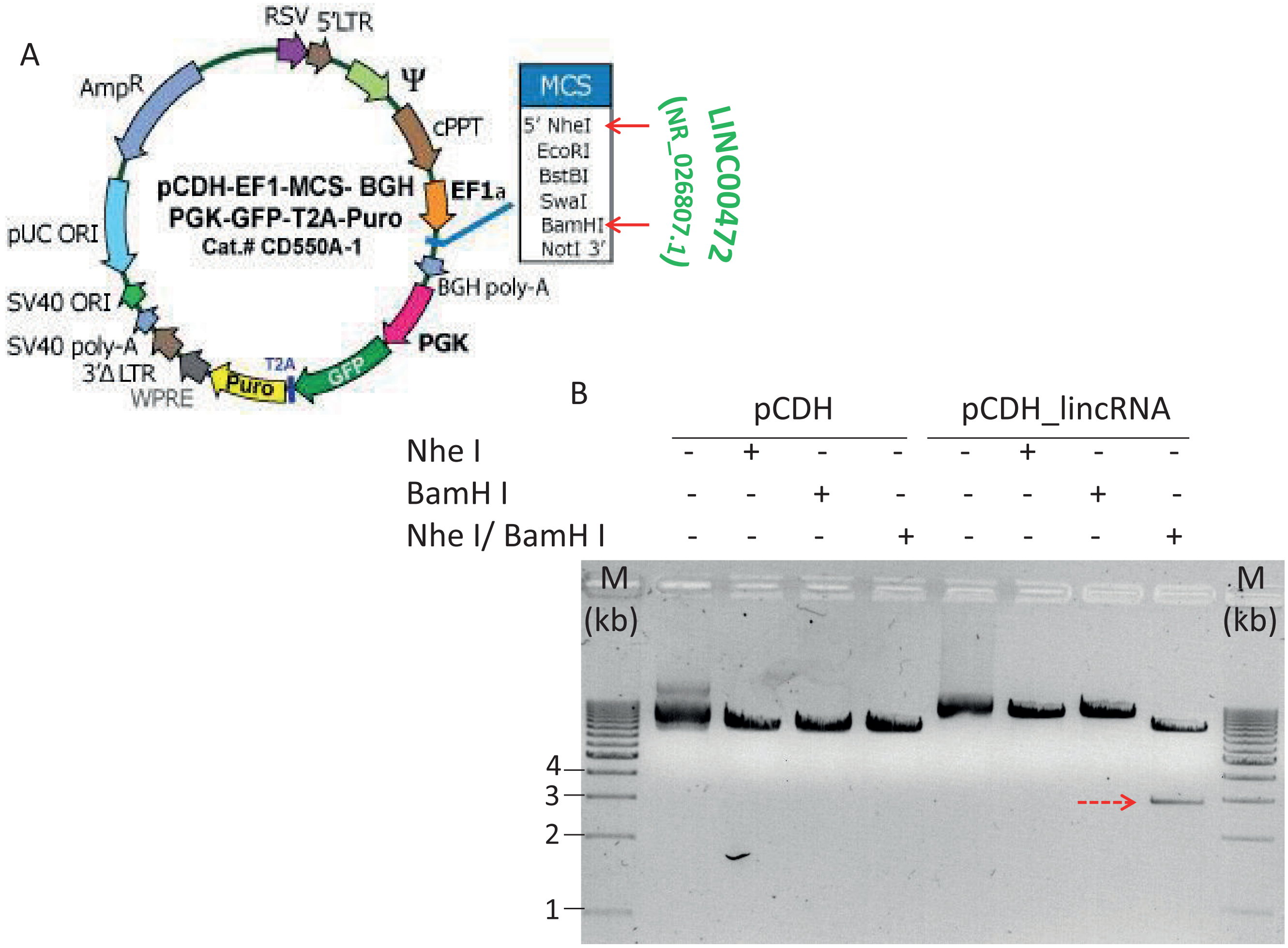

Supplementary Figure S4: *LINC00472* construct and verification. A. A *LINC00472* transcript (2933bp, NR_026807.1) was assembled from several EST clones (EHS1001-207275390, EHS1001-207498495, EHS1001-207533792, EHS1001-207590772, EHS1001-210281579, and EHS1001-211231922 from Thermo Scientific Open Biosystems). Two restriction sites, Nhe1 and BamH1, were incorporated into the 5′ and 3′ end of the transcript, respectively. The entire sequence was inserted into a lentiviral expression vector, pCDH-EF1-MCS-pA-PGK-copGFP-T2A-Puro (System Biosciences). In this vector, EF1a promoter drives the expression of *LINC00472* transcript and PGK promoter directs a report gene, GFP, serving as a transfection control. **B.** Restriction enzyme digestion was performed to the control vector, pCDH and the vector with *LINC00472* transcript, pCDH_*LINC00472*. Agarose gel electrophoresis showed that a single digestion produced only one DNA fragment both in pCDH and pCDH_*LINC00472*, and a double digestion on pCDH_*LINC00472* produced an additional DNA fragment at about a 3 kb position (red arrow), indicating the presence of the inserted transcript, which didn't show in the double digestion of pCDH.

UCSC Genome Browser on Human Feb. 2009 (GRCh37/hg19) Assembly

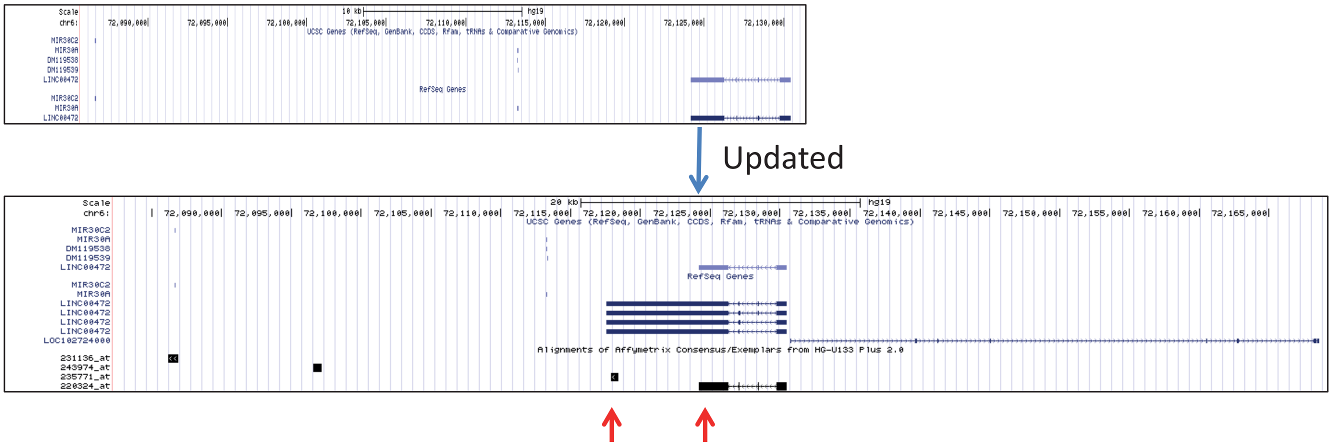

Supplementary Figure S5: Updated *LINC00472* transcripts. Recently *LINC00472* transcripts have been updated to include NR_121612.1, NR_026807.2, NR_121613.1 and NR_121614.1. The black blocks show the targeted areas of the probes designed in Affymetrix array HG-U133 plus 2, and the red arrows indicate the probes from which we used expression data for analysis.

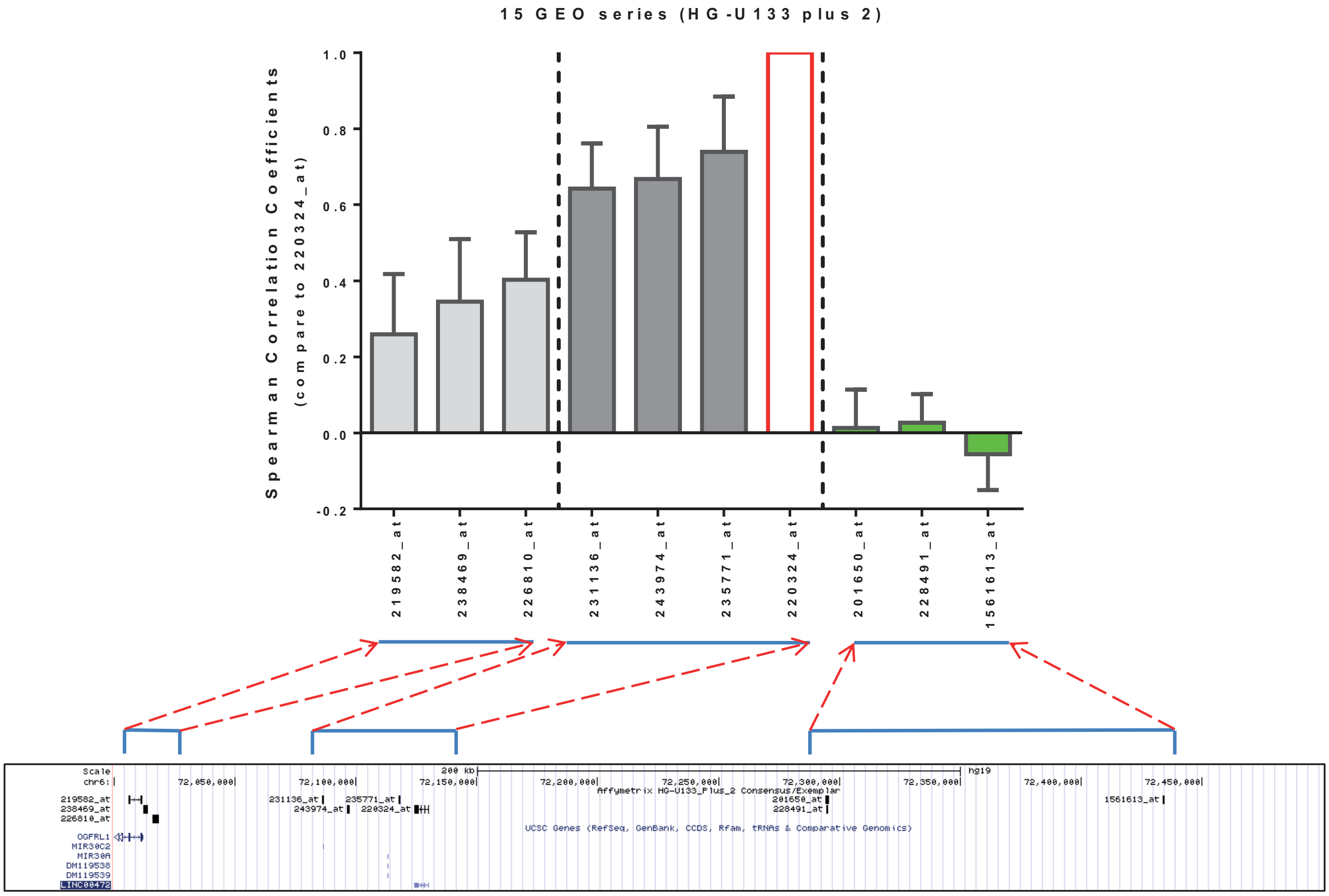

Supplementary Figure S6: Multiple probes in HG-U133 plus 2 array for *LINC00472*. The probe, 235771_at (in dark grey), covers the updated *LINC00472* transcripts as probe 220324_at (red rectangle) does. The expression intensity of these two probes is strongly correlated in 15 GEO datasets. The values in y-axis show the correlation coefficients between expression intensity from different probes and that from probe 220324_at. The grey and green bars represent the probes further away from probe 220324_at.

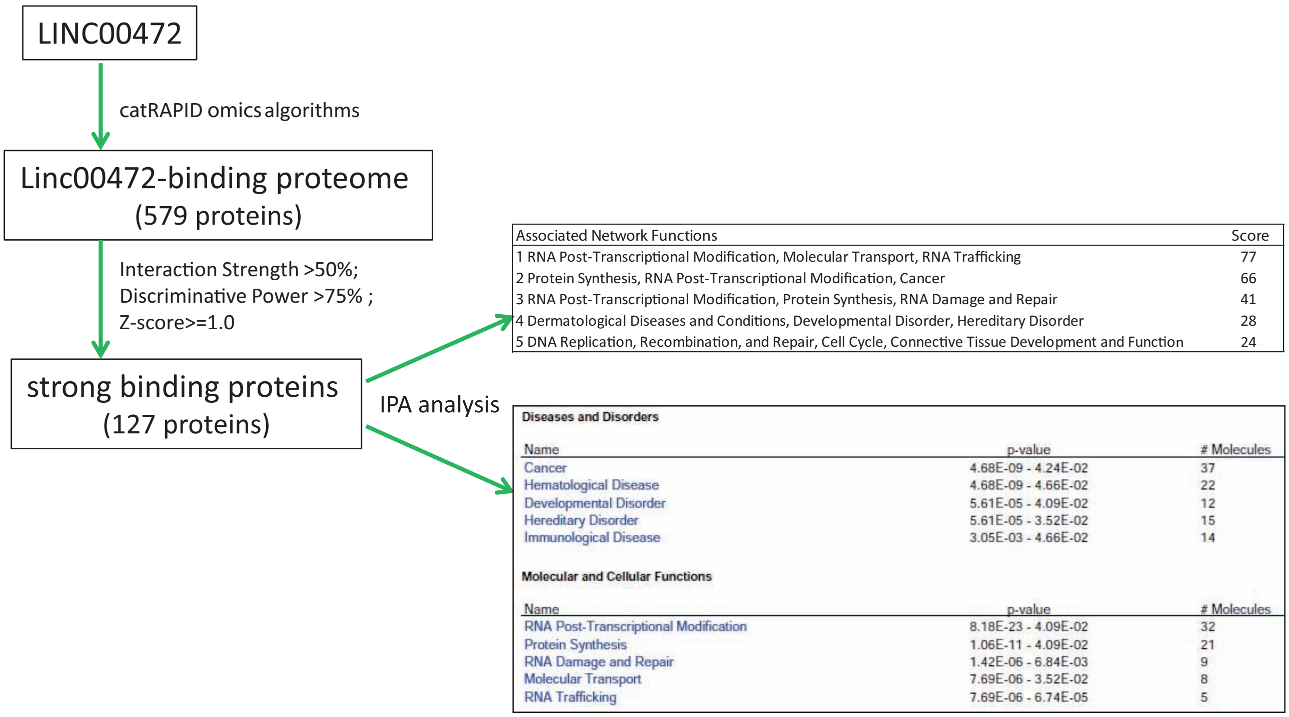

Associated Network Functions	Score
1 RNA Post-Transcriptional Modification, Molecular Transport, RNA Trafficking	77
2 Protein Synthesis, RNA Post-Transcriptional Modification, Cancer	66
3 RNA Post-Transcriptional Modification, Protein Synthesis, RNA Damage and Repair	41
4 Dermatological Diseases and Conditions, Developmental Disorder, Hereditary Disorder	28
5 DNA Replication, Recombination, and Repair, Cell Cycle, Connective Tissue Development and Function	24

Diseases and Disorders

Name	p-value	# Molecules
Cancer	4.68E-09 - 4.24E-02	37
Hematological Disease	4.68E-09 - 4.66E-02	22
Developmental Disorder	5.61E-05 - 4.09E-02	12
Hereditary Disorder	5.61E-05 - 3.52E-02	15
Immunological Disease	3.05E-03 - 4.66E-02	14

Molecular and Cellular Functions

Name	p-value	# Molecules
RNA Post-Transcriptional Modification	8.18E-23 - 4.09E-02	32
Protein Synthesis	1.06E-11 - 4.09E-02	21
RNA Damage and Repair	1.42E-06 - 6.84E-03	9
Molecular Transport	7.69E-06 - 3.52E-02	8
RNA Trafficking	7.69E-06 - 6.74E-05	5

Supplementary Figure S7: Prediction of *LINC00472*-binding proteins. *cat*RAPID *omics* algorithm predicts that *LINC00472* has 579 binding proteins, in which 127 proteins have strong interaction propensities. Ingenuity Core Analysis with these proteins shows the top disease associated with *LINC00472*-binding proteins is cancer, and the top associated network of *LINC00472*-binding proteins is RNA post-transcriptional modification.

Supplementary Figure S8A–S8C: Top 3 networks associated with *LINC00472*-binding proteins. IPA analysis showed 3 networks (S8A, S8B and S8C) relevant to *LINC00472*. The solid lines denote a robust correlation with partner proteins, and dashed lines indicate statistically significant but less frequent correlations. The grey nodes indicate the *LINC00472*-binding proteins predicted by *catRAPID*; the red color represents the proteins associated with RNA post-transcriptional modifications, Protein Synthesis and Cancer; the un-colored nodes indicate additional proteins of this network that were not spotted by the prediction. The shape of the nodes denote the protein's function: enzymes (diamond); nuclear receptors (rectangle); transcription regulators (oval); cytokines (square); transporter (trapezoid); and others (circle). The breast cancer-related biomarkers found in the networks are marked with BM in green.

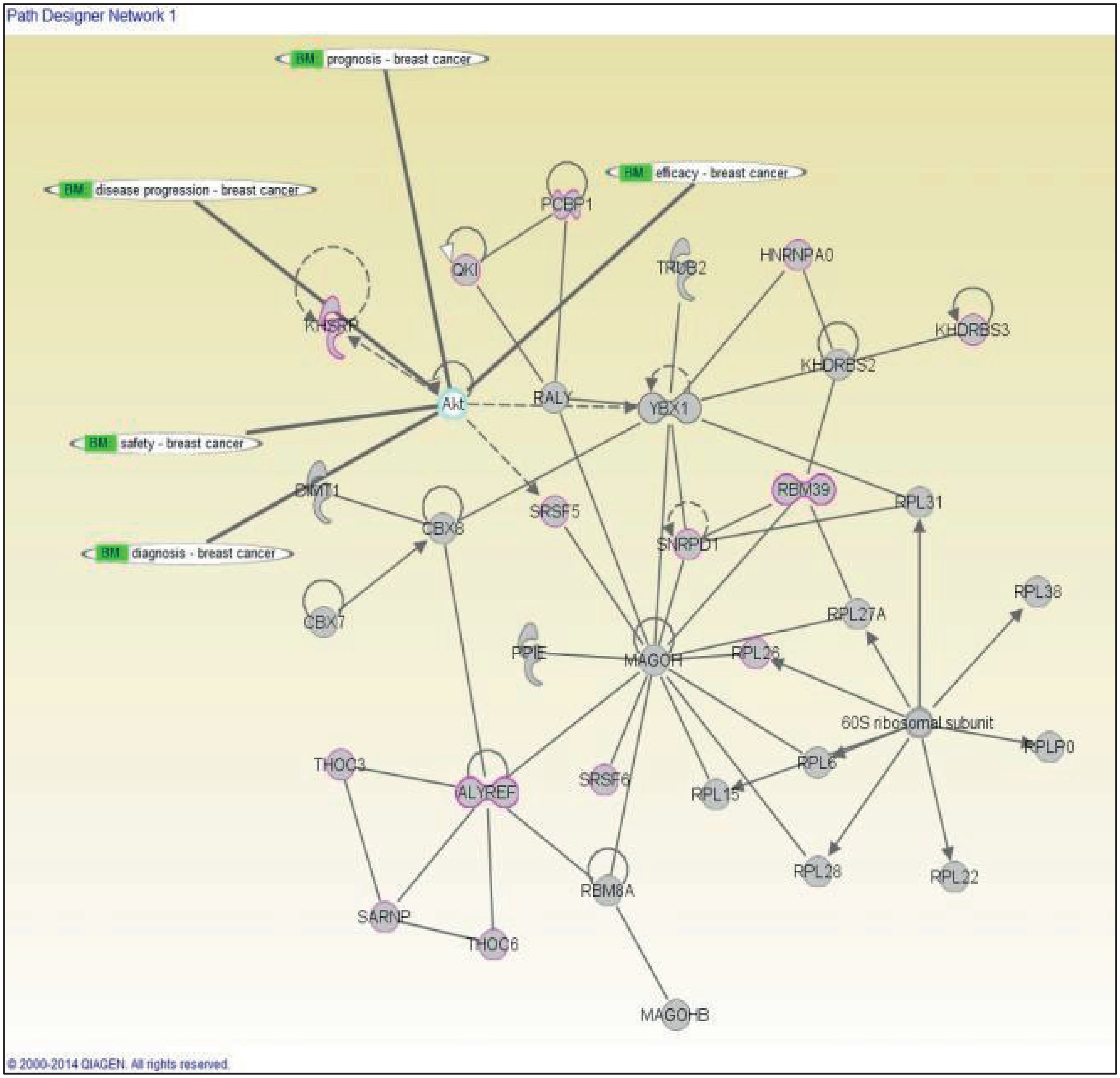

(*Continued*)

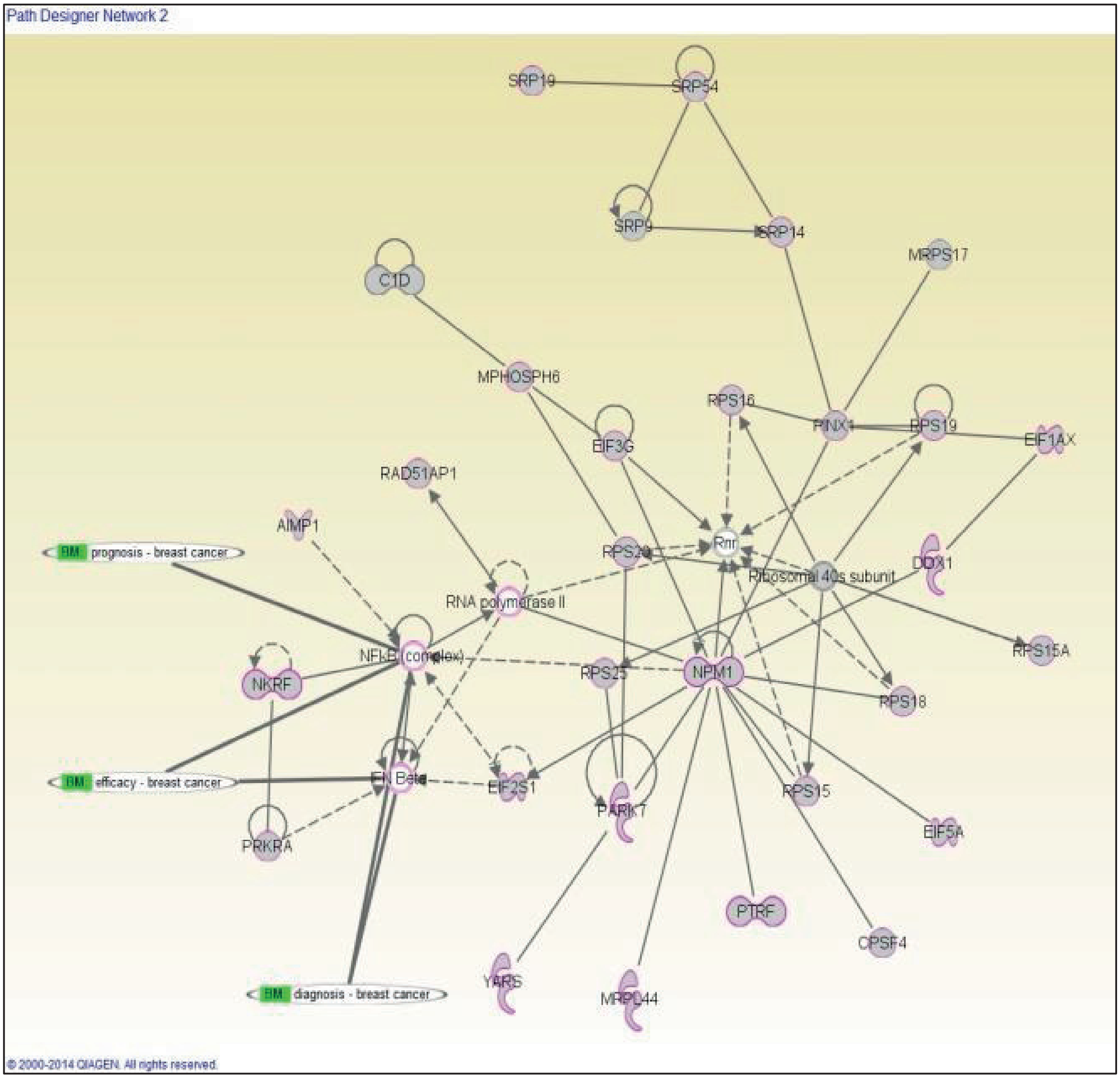
Path Designer Network 2
SRP19
SRP54
SRP9
SRP14
MRPS17
C1D
MPHOSPH6
RPS16
PNX1
RPS19
EIF1AX
EIF3G
RAD51AP1
AIMP1
BM prognosis - breast cancer
Rnr
RPS20
Ribosomal 40s subunit
DDX1
RNA polymerase II
NFkB (complex)
RPS15A
NKRF
RPS25
NPM1
RPS18
BM efficacy - breast cancer
IFN Beta
EIF2S1
PARK7
RPS15
EIF5A
PRKRA
PTRF
CPSF4
YARS
MRPL44
BM diagnosis - breast cancer
© 2000-2014 QIAGEN. All rights reserved.

(*Continued*)

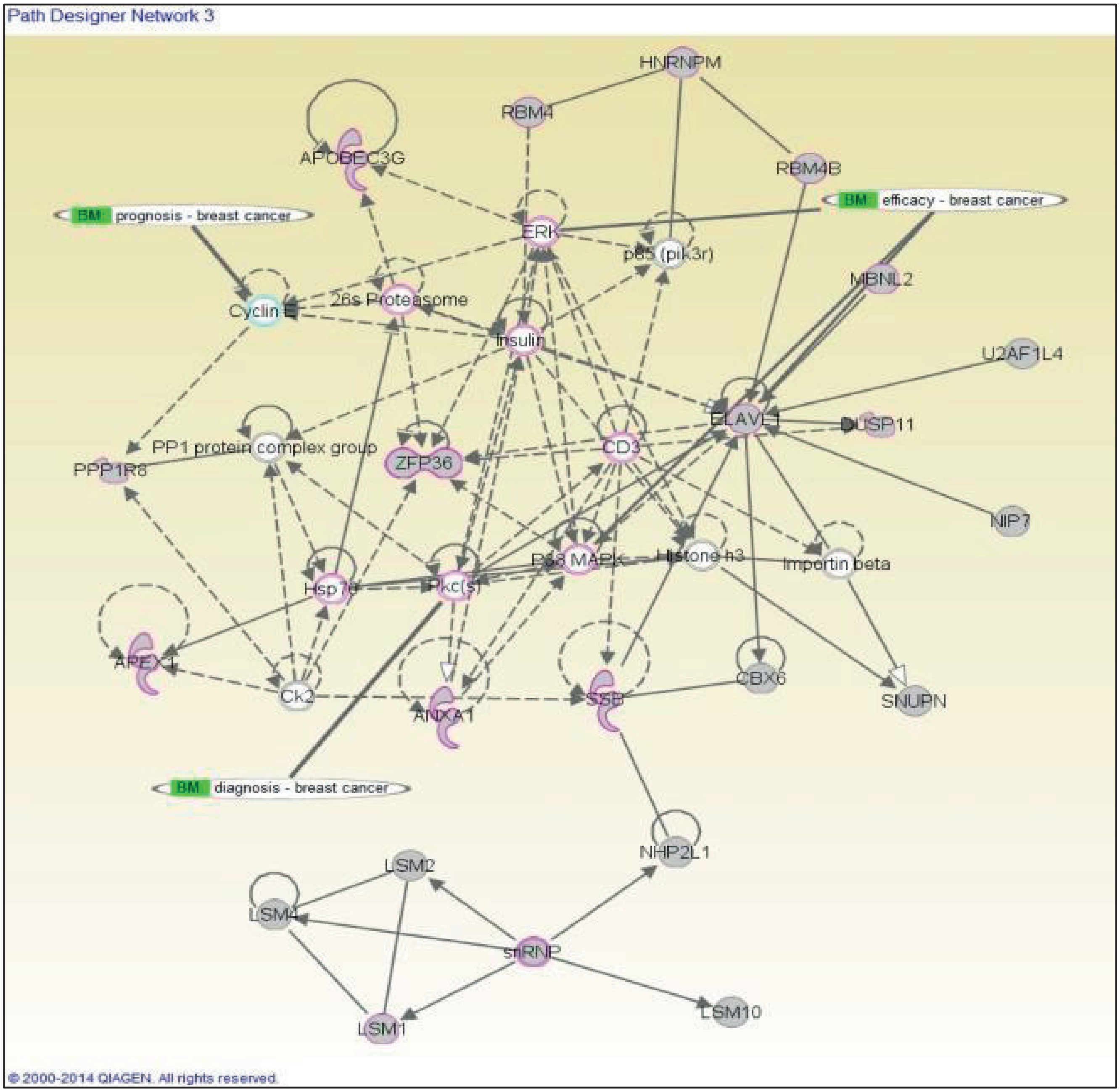
Path Designer Network 3
HNRNPM
RBM4
APOBEC3G
RBM4B
BM prognosis - breast cancer
BM efficacy - breast cancer
ERK
p85 (pik3r)
MBNL2
Cyclin E
26s Proteasome
Insulin
U2AF1L4
ELAVL1
DUSP11
PP1 protein complex group
PPP1R8
ZFP36
CD3
NIP7
P38 MAPK
Histone h3
Importin beta
Hsp70
Pkc(s)
APEX1
CBX6
Ck2
ANXA1
SSB
SNUPN
BM diagnosis - breast cancer
NHP2L1
LSM2
LSM4
snRNP
LSM1
LSM10
© 2000-2014 QIAGEN. All rights reserved.

Supplementary Table S1. Summary of Publically Available Breast Cancer Microarray Datasets

GEO Series (Ref)	Country	Case Number	Clinical Information							Platform	Analysis Method
			ER	PR	Her2	Grade	Nodal status	Molecular subtype	Survival outcomes		
GSE2034[1]	Netherlands	286	Yes						DMFS[a]	GPL96[f]	log2 transformed MAS5.0 signal intensity
GSE2603[2]	USA	99	Yes	Yes	Yes		Yes		LMFS[b]; BMFS[c]	GPL96	log2 transformed MAS5.0 signal intensity
GSE3494[3]	Sweden	251	Yes	Yes		Yes	Yes		DFS[d]	GPL96	log2 transformed MAS5.0 signal intensity
GSE4922[4]	Sweden, Singapore	289	Yes			Yes	Yes		DFS	GPL96	log2 transformed MAS5.0 signal intensity
GSE5460[5]	USA	129	Yes		Yes	Yes	Yes			GPL570[g]	dChip signal intensity
GSE6532[6–8]	UK, Sweden	87		Yes		Yes	Yes		DFS	GPL570	RMA log 2 expression levels
GSE7390[9]	France, UK, Sweden	198	Yes			Yes			DMFS, OS[e]	GPL96	log2 transformed MAS5.0 signal intensity
GSE11121[10]	Germany	200				Yes			DMFS	GPL96	log2 transformed MAS5.0 signal intensity
GSE18864[11–13]	USA	84	Yes	Yes	Yes	Yes				GPL570	RMA log 2 expression levels
GSE19615[12]	USA	115	Yes	Yes	Yes	Yes	Yes		DFS	GPL570	dChip signal intensity
GSE20194[14,15]	USA	278	Yes	Yes	Yes	Yes				GPL96	log2 transformed MAS5.0 signal intensity
GSE20271[16]	USA	178	Yes	Yes	Yes	Yes				GPL96	log10 transformed dChip signal intensity
GSE20685[17]	Taiwan	327							OS	GPL570	log2 transformed MAS5.0 signal intensity
GSE21653[18,19]	France	266	Yes	Yes		Yes		Yes	DFS	GPL570	RMA log 2 expression levels
GSE23177[20]	Belgium	116					Yes			GPL570	log2 transformed MAS5.0 signal intensity

(*Continued*)

GEO Series (Ref)	Country	Case Number	Clinical Information							Platform	Analysis Method
			ER	PR	Her2	Grade	Nodal status	Molecular subtype	Survival outcomes		
GSE23720[18,19]	France	197	Yes	Yes						GPL570	RMA log 2 expression levels
GSE23988[21]	USA	61	Yes			Yes	Yes			GPL96	log2 transformed dChip signal intensity
GSE24185[22]	USA	103	Yes	Yes	Yes	Yes	Yes			GPL96	dChip signal intensity
GSE25055[23]	USA	310	Yes	Yes	Yes	Yes	Yes	Yes	DMFS	GPL96	log2 transformed MAS5.0 signal intensity
GSE25065[23]	USA	198	Yes	Yes	Yes	Yes	Yes	Yes	DMFS	GPL96	log2 transformed MAS5.0 signal intensity
GSE31448[18,19]	France	353	Yes	Yes		Yes		Yes	DFS	GPL570	RMA log 2 expression levels
GSE42568[24]	Ireland	104	Yes			Yes	Yes		DMFS, DFS, OS	GPL570	Log2 GC-RMA signal intensity
GSE47109[25]	USA	246	Yes			Yes				GPL570	log2 transformed MAS5.0 signal intensity
GSE48390[26]	Taiwan	81	Yes		Yes			Yes	DFS	GPL570	RMA log 2 expression levels
Turin_study	Italy	348	Yes	Yes		Yes	Yes	Yes	DFS, OS	Q-PCR	Expression index
**Total**:		**4904**									

[a]DMFS - Distance Metastasis Free Survival
[b]LMFS - Lung Metastasis Free Survival
[c]BMFS - Bone Metastasis Free Survival
[d]DFS - Disease Free Survival
[e]OS - Overall Survival
[f]GPL96 - Affymetrix Human Genome U133A Array (HG-U133A)
[g]GPL570 - Affymetrix Human Genome U133 Plus 2.0 Array (HG-U133 Plus 2)

REFERENCES IN SUPPLEMENTARY TABLE S1

1. Wang Y, Klijn JG, Zhang Y, et al. Gene-expression profiles to predict distant metastasis of lymph-node-negative primary breast cancer. Lancet. 2005; 365:671–9.
2. Minn AJ, Gupta GP, Siegel PM, et al. Genes that mediate breast cancer metastasis to lung. Nature. 2005; 436:518–24.
3. Miller LD, Smeds J, George J, et al. An expression signature for p53 status in human breast cancer predicts mutation status, transcriptional effects, and patient survival. Proceedings of the National Academy of Sciences of the United States of America. 2005; 102:13550–5.
4. Ivshina AV, George J, Senko O, et al. Genetic reclassification of histologic grade delineates new clinical subtypes of breast cancer. Cancer research. 2006; 66:10292–301.
5. Lu X, Lu X, Wang ZC, Iglehart JD, Zhang X, Richardson AL. Predicting features of breast cancer with gene expression patterns. Breast cancer research and treatment. 2008; 108:191–201.
6. Loi S, Haibe-Kains B, Desmedt C, et al. Definition of clinically distinct molecular subtypes in estrogen receptor-positive breast carcinomas through genomic grade. Journal of clinical oncology : official journal of the American Society of Clinical Oncology. 2007; 25:1239–46.
7. Loi S, Haibe-Kains B, Majjaj S, et al. PIK3CA mutations associated with gene signature of low mTORC1 signaling and better outcomes in estrogen receptor-positive breast cancer. Proceedings of the National Academy of Sciences of the United States of America. 2010; 107:10208–13.
8. Loi S, Haibe-Kains B, Desmedt C, et al. Predicting prognosis using molecular profiling in estrogen receptor-positive breast cancer treated with tamoxifen. BMC genomics. 2008; 9:239.
9. Desmedt C, Piette F, Loi S, et al. Strong time dependence of the 76-gene prognostic signature for node-negative breast cancer patients in the TRANSBIG multicenter independent validation series. Clinical cancer research: an official journal of the American Association for Cancer Research. 2007; 13:3207–14.
10. Schmidt M, Bohm D, von Torne C, et al. The humoral immune system has a key prognostic impact in node-negative breast cancer. Cancer research. 2008; 68:5405–13.
11. Silver DP, Richardson AL, Eklund AC, et al. Efficacy of neoadjuvant Cisplatin in triple-negative breast cancer. Journal of clinical oncology: official journal of the American Society of Clinical Oncology. 2010; 28:1145–53.
12. Li Y, Zou L, Li Q, et al. Amplification of LAPTM4B and YWHAZ contributes to chemotherapy resistance and recurrence of breast cancer. Nature medicine. 2010; 16:214–8.
13. Juul N, Szallasi Z, Eklund AC, et al. Assessment of an RNA interference screen-derived mitotic and ceramide pathway metagene as a predictor of response to neoadjuvant paclitaxel for primary triple-negative breast cancer: a retrospective analysis of five clinical trials. The lancet oncology. 2010; 11:358–65.
14. Popovici V, Chen W, Gallas BG, et al. Effect of training-sample size and classification difficulty on the accuracy of genomic predictors. Breast cancer research: BCR. 2010; 12:R5.
15. Shi L, Campbell G, Jones WD, et al. The MicroArray Quality Control (MAQC)-II study of common practices for the development and validation of microarray-based predictive models. Nature biotechnology. 2010; 28:827–38.
16. Tabchy A, Valero V, Vidaurre T, et al. Evaluation of a 30-gene paclitaxel, fluorouracil, doxorubicin, and cyclophosphamide chemotherapy response predictor in a multicenter randomized trial in breast cancer. Clinical cancer research: an official journal of the American Association for Cancer Research. 2010; 16:5351–61.
17. Kao KJ, Chang KM, Hsu HC, Huang AT. Correlation of microarray-based breast cancer molecular subtypes and clinical outcomes: implications for treatment optimization. BMC cancer. 2011; 11:143.
18. Sabatier R, Finetti P, Adelaide J, et al. Down-regulation of ECRG4, a candidate tumor suppressor gene, in human breast cancer. PloS one. 2011; 6:e27656.
19. Sabatier R, Finetti P, Cervera N, et al. A gene expression signature identifies two prognostic subgroups of basal breast cancer. Breast cancer research and treatment. 2011; 126:407–20.
20. Smeets A, Daemen A, Vanden Bempt I, et al. Prediction of lymph node involvement in breast cancer from primary tumor tissue using gene expression profiling and miRNAs. Breast cancer research and treatment. 2011; 129:767–76.
21. Iwamoto T, Bianchini G, Booser D, et al. Gene pathways associated with prognosis and chemotherapy sensitivity in molecular subtypes of breast cancer. Journal of the National Cancer Institute. 2011; 103:264–72.
22. Creighton CJ, Sada YH, Zhang Y, et al. A gene transcription signature of obesity in breast cancer. Breast cancer research and treatment. 2012; 132:993–1000.
23. Hatzis C, Pusztai L, Valero V, et al. A genomic predictor of response and survival following taxane-anthracycline chemotherapy for invasive breast cancer. JAMA: the journal of the American Medical Association. 2011; 305:1873–81.
24. Clarke C, Madden SF, Doolan P, et al. Correlating transcriptional networks to breast cancer survival: a large-scale coexpression analysis. Carcinogenesis. 2013; 34:2300–8.
25. D'Alfonso TM, van Laar RK, Vahdat LT, et al. BreastPRS is a gene expression assay that stratifies intermediate-risk Oncotype DX patients into high- or low-risk for disease recurrence. Breast cancer research and treatment. 2013; 139:705–15.
26. Huang CC, Tu SH, Lien HH, et al. Concurrent gene signatures for han chinese breast cancers. PloS one. 2013; 8:e76421.
